# Chloroplast gene expression level is negatively correlated with evolutionary rates and selective pressure while positively with codon usage bias in *Ophioglossum vulgatum* L

**DOI:** 10.1186/s12870-022-03960-8

**Published:** 2022-12-13

**Authors:** Jing Hao, Yingyi Liang, Jingyao Ping, Jinye Li, Wanxin Shi, Yingjuan Su, Ting Wang

**Affiliations:** 1 College of Life Sciences, South China Agricultural University, Guangzhou, 510642 China; 2 School of Life Sciences, Sun Yat-sen University, Guangzhou, 510275 China; 3 Research Institute of Sun Yat-sen University in Shenzhen, Shenzhen, 518057 China

**Keywords:** *Ophioglossum vulgatum*, Chloroplast genome, Gene expression level, Evolutionary rate, Selective pressure, Codon usage bias

## Abstract

**Background:**

Characterization of the key factors determining gene expression level has been of significant interest. Previous studies on the relationship among evolutionary rates, codon usage bias, and expression level mostly focused on either nuclear genes or unicellular/multicellular organisms but few in chloroplast (cp) genes. *Ophioglossum vulgatum* is a unique fern and has important scientific and medicinal values. In this study, we sequenced its cp genome and transcriptome to estimate the evolutionary rates (*dN* and *dS*), selective pressure (*dN*/*dS*), gene expression level, codon usage bias, and their correlations.

**Results:**

The correlation coefficients between *dN, dS,* and *dN*/*dS*, and Transcripts Per Million (TPM) average values were -0.278 (*P* = 0.027 < 0.05), -0.331 (*P* = 0.008 < 0.05), and -0.311 (*P* = 0.013 < 0.05), respectively. The codon adaptation index (CAI) and tRNA adaptation index (tAI) were significantly positively correlated with TPM average values (*P* < 0.05).

**Conclusions:**

Our results indicated that when the gene expression level was higher, the evolutionary rates and selective pressure were lower, but the codon usage bias was stronger. We provided evidence from cp gene data which supported the E-R (E stands for gene expression level and R stands for evolutionary rate) anti-correlation.

**Supplementary Information:**

The online version contains supplementary material available at 10.1186/s12870-022-03960-8.

## Background

Evolutionary rate of different proteins varies greatly, and the search for the determinants of this rate variation has been a central question in evolutionary biology [1]. The protein evolutionary rate is suggested related to many variables, of which one of the best predictors is gene expression level [2, 3]. Previous studies have proposed an anticorrelation relationship between gene expression level and evolutionary rate (E-R anticorrelation) [4, 5]. And the E-R anticorrelation has been observed in *Brassica* [6], *Arachis* [7], and *Pyrus* orthologous genes [8] as well as *Brassica napus* vernalization-pathway genes [3]. However, the E-R anticorrelation has not always been lent support. For example, Hunt et al. [9] have observed that gene expression level is positively correlated with the protein evolutionary rate at both intra- and interspecific level in *Solenopsis*. Moreover, Feyertag et al. [10] have also noted that highly expressed N-glycoproteins evolve faster. Therefore, the E-R anticorrelation remains to be tested.

Codon usage bias means that synonymous codons encoding the same amino acid are used at different frequencies [11]. Codon usage bias has been found to strongly correlate with gene expression level [12, 13]. The use of synonymous codons varies with different genomes and different genes within the same genome [14, 15]. Generally, codon usage bias is thought to be maintained by a balance between selection (optimal codon) and mutation together with drift (nonoptimal codons) (selection-mutation-drift-theory) [11, 16–18]. Codons of highly expressed genes are used more frequently and selection may be stronger, so that it produces greater bias [11]. Natural selection may act on gene codon usage by selection at a single nucleotide site (the site is independent of its protein coding function) or dependent on the amino acid coding of codons [19]. The latter role results in codon adaptation that increase translation efficiency [19]. In addition, the usage of preferred codon may improve the accuracy of translation, which is closely related to the tRNA abundance and expression level [20–22]. tRNAs with preferred anticodons may be associated with more precise amino acid acylation, increasing protein synthesis rate [20]. Moreover, there are several other factors enable to affect codon bias. For example, mRNA secondary structure at the 5’ end has an indirect influence on codon usage frequency [23]; longer protein tends to have stronger codon usage bias, as selection favors codons that may improve translation accuracy and is greater on longer protein-coding genes [24].

**Fig. 1:**
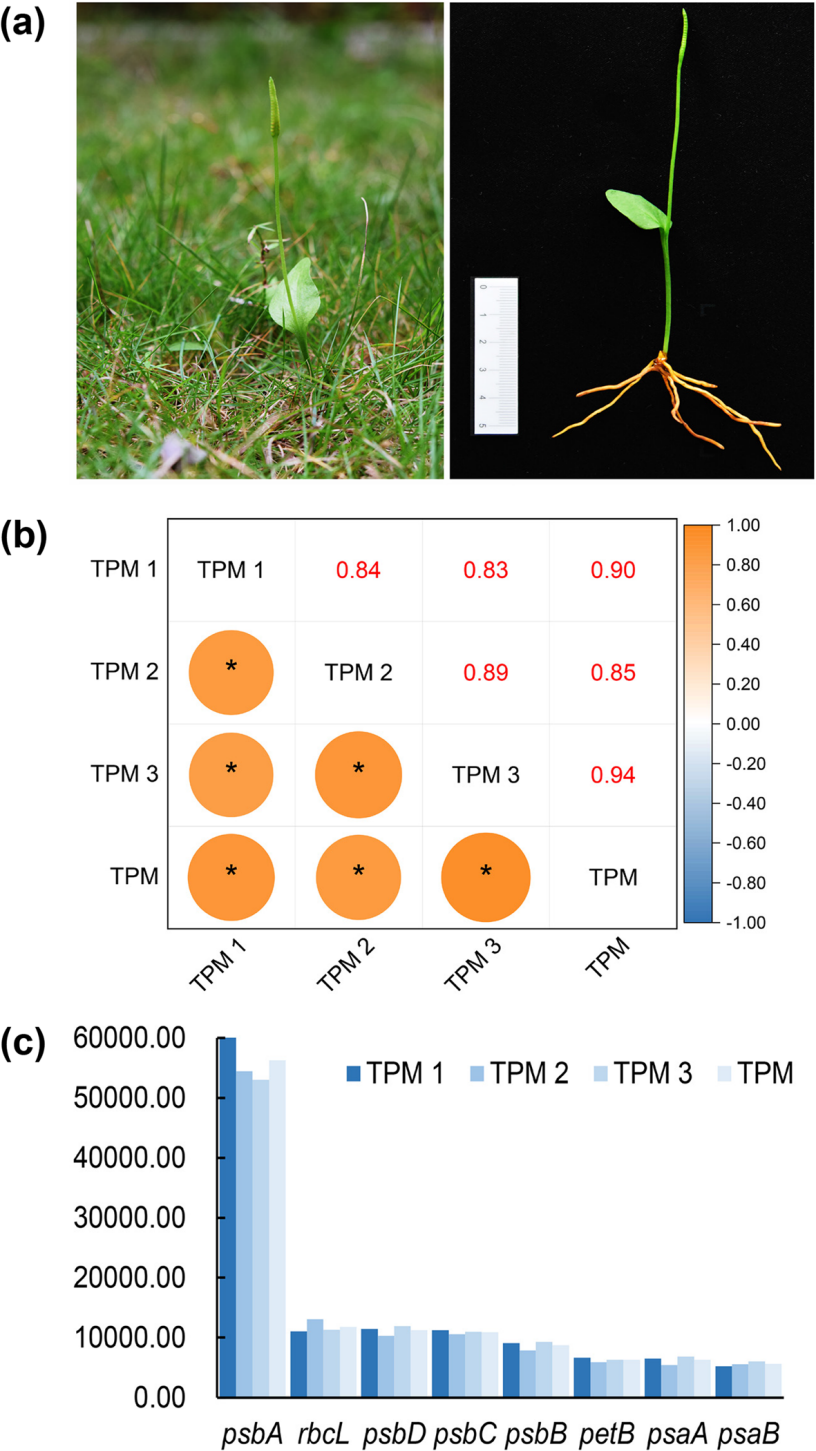
Morphological characteristics and TPM (the Transcripts Per Million) values of chloroplast genes in *O. vulgatum*. **(a)** Habitat and morphological characteristics of *O. vulgatum* (photos were taken by ourselves). **(b)** Heat map of Spearman’s rank correlation coefficient for all genes’ TPM values of three biological repeats (the numbers on the upper triangular are correlation coefficients; the TPM1, TPM2, and TPM3 are the TPM value of three repeats, the 'TPM' is the mean value of biological repeats, the same as below; * represents significant at the 0.05 level). **(c)** TPM values of highly expressed genes in chloroplast

Previous studies on the relationship among evolutionary rate, codon usage bias, and expression level have mostly focused on nuclear genes or unicellular/multicellular organisms. By contrast, chloroplast (cp) is a semi-autonomous organelle with its own genome. In comparison with the nuclear genome, cp genome is small in size, simple and conserved in structure, and moderate in nucleotide evolution rate. Cp genome has been widely used in taxonomy, phylogenetic and evolutionary studies [25–27]. But whether the expression of cp genes is consistent or not with the E-R anticorrelation remains to be tested. *O. vulgatum* is a rare and ancient fern with high medicinal value [28–30]. The plant is 10–30 cm high with a huge number of chromosomes (2n = 240–1140), tending to live in shaded forests and wet meadows [30–34] (Fig. 1a).

More recently, we have sequenced the complete cp genome of *O. vulgatum* (GenBank No. MZ066610) [29]. Here we have further sequenced its transcriptome. Based on the data, in this study we have firstly analyzed the correlationships between gene expression level and evolutionary rates, selective pressure, and codon usage bias. We have observed that cp gene expression level is negatively correlated with evolutionary rates and selective pressure but positively with codon usage bias in *O. vulgatum*. This provides new evidence for understanding the evolution of fern cp genes.

## Results

### RNA sequencing data

Illumina high-throughput sequencing results were converted into raw reads after base calling by using CASAVA. High-quality clean reads were obtained after filtering raw reads. Paired reads were removed when N content in any read exceeded 10% of the read base number and the number of low-quality (Quality values ≤ 5) bases in any read exceeded 50% of the read base number. Our sequence data were deposited in the National Center for Biotechnology Information (NCBI) Sequence Read Archive (SRA) with accession number PRJNA789441. Statistics of the sequencing data is shown in Table 1. The average GC, Q20 and Q30 of the three samples were 46.17%, 97.56%, and 93.70%, respectively. Both Q20 and Q30 reached more than 90%.

**Table 1:** RNA sequencing data and the number of RNA reads mapped with cp genes

Sample	Reads Number	Total Base	GC	Q20	Q30	RNA reads Number (mapped with cp genes)
repeat1	22,745,713	6,823,713,900	46.35%	98.07%	94.71%	93, 463
	22,745,713		46.11%	97.44%	93.41%	
repeat2	22,757,747	6,827,324,100	46.41%	97.93%	94.26%	185, 302
	22,757,747		46.17%	96.90%	92.47%	
repeat3	25,884,993	7,765,497,900	46.12%	98.04%	94.69%	109, 383
	25,884,993		45.83%	97.00%	92.64%	

### The expression level of *O. vulgatum* chloroplast genes

The Transcripts Per Million (TPM) values of all cp protein-coding genes of *O. vulgatum* were calculated. The number of RNA reads mapped with cp genes is shown in Table 1. A heat map of Spearman’s rank correlation coefficient established based on three repeats is shown as Fig. 1b. The correlation coefficient between the TPM value of three repeats and its arithmetic mean was more than 0.83. Top 10% genes in terms of mean TPM were taken as highly expressed [15]. Genes with high expression level were *psaA, psaB, psbA*, *psbB, psbC*, *psbD*, *petB*, and *rbcL* (Fig. 1c, Additional file 2). Among them, *psbA* had the highest TPM. All high expression genes were associated with the photosynthetic system.

### *dN*,* dS*, and *dN/dS* values of conserved chloroplast genes

**Fig. 2:**
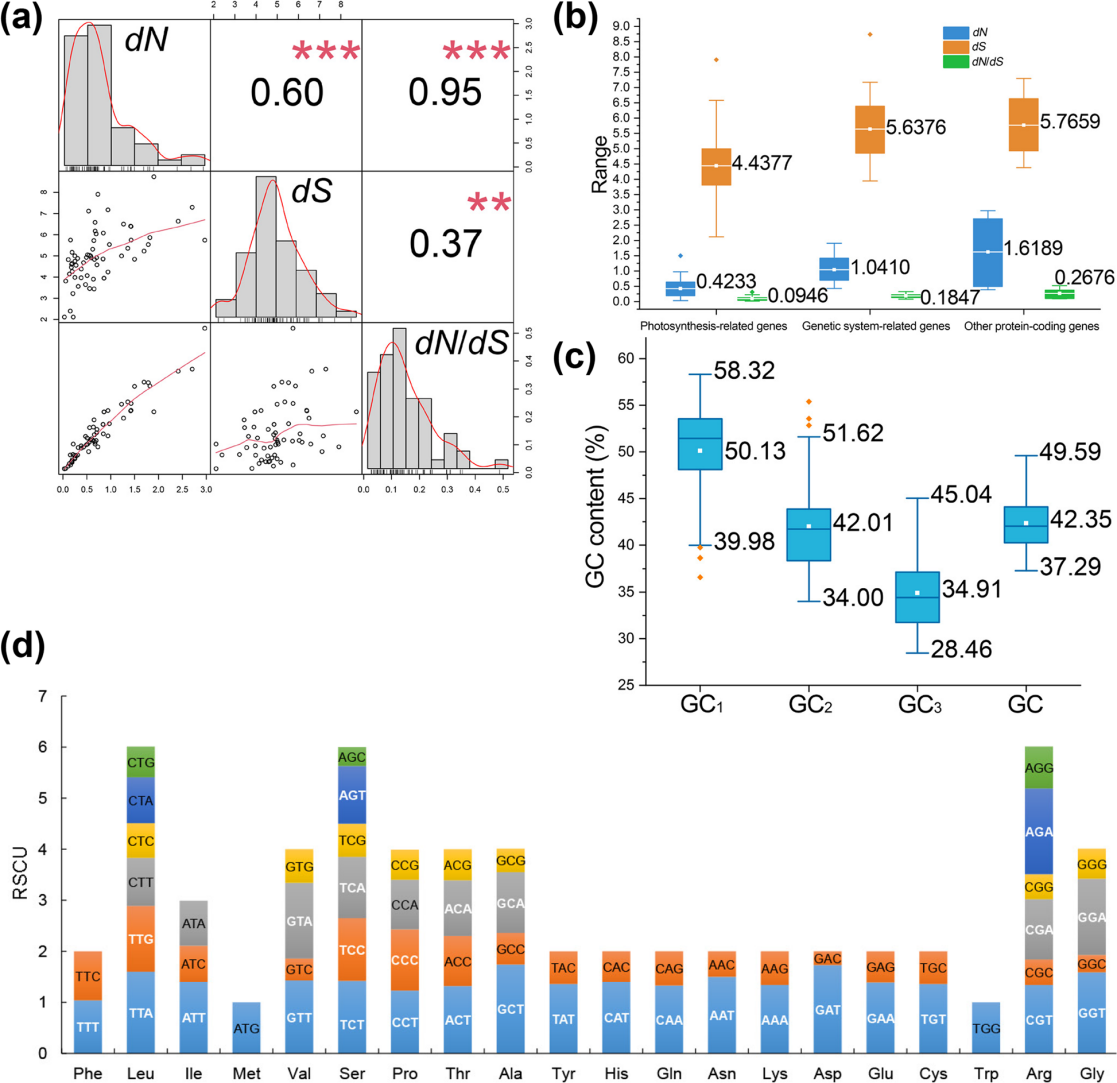
*dN* (nonsynonymous substitution rate), *dS* (synonymous substitution rate), *dN*/*dS* (selective pressure, *ω*) and codon usage bias analysis results. **(a)** Distribution and correlation (Spearman’s rank correlation coefficient) of *dN*, *dS*, and *dN*/*dS* in conserved chloroplast genes (** represents *P* < 0.01. *** represents *P* < 0.001). **(b)** The boxplot of *dN*, *dS*, and *dN*/*dS* distribution of chloroplast genes with three different functions (photosynthesis-related genes, genetic system-related genes and other protein-coding genes). **(c)** Boxplot of GC content variation in different codon positions. GC content at the first base (GC_1_); GC content at the second base position (GC_2_); the GC content at the third base position of codons (GC_3_); the overall GC content (GC). The orange dots represent outliers and the white represent mean values. **(d)** RSCU (relative synonymous codon usage) value of amino acid and codons. Different colors of columns represent different codons encoding the same amino acid. The Codons in white font were the high-frequency codons

The evolutionary rates of protein-coding genes included synonymous substitution rate (*dS*) and nonsynonymous substitution rate (*dN*). The value of *dN*/*dS* (*ω*) was used to measure selective pressure (with *ω* < 1, *ω* = 1, and *ω* > 1 indicating negative, neutral, and positive selection, respectively) [35]. CDS (Protein coding sequence) sequences of a total of 64 conservative genes were extracted for manual correction. Phylogenetic results showed that the divergence time of Ophioglossaceae was later than Equisetaceae but earlier than other selected species (Additional file 3), which is consistent with the PPG I system [36]. The mean values of *dN*, *dS*, and *dN*/*dS* of the 64 genes were 0.7664, 4.9955, and 0.1445, respectively (Table 2, Additional file 4). Then the 64 conservative genes were divided into three functional groups: photosynthesis-related genes, genetic system-related genes, and other functional protein-coding genes [37, 38] (Additional file 5). The *dN* value was from 0.0301 (*psbA*) to 2.9696 (*ycf2*); the *dS* value was from 2.1131 (*psbL*) to 8.7323 (*rpl23*); and the *dN*/*dS* value was from 0.0142 (*psbA*) to 0.5173 (*ycf2*). The *dN*/*dS* value was significantly positively correlated with *dN* (*P* < 0.001) and *dS* (*P* < 0.01) (Fig. 2a). The positive correlation coefficient between *dN*/*dS* and *dN* (0.95) was greater than between *dN*/*dS* and *dS* (0.37). The mean value of *dN*/*dS* of photosynthesis-related genes, genetic system genes, and other functional protein-coding genes were 0.0946, 0.1847 and 0.2676, respectively (Fig. 2b). The mean value of *dN, dS,* and *dN*/*dS* of photosynthetic system genes was the lowest, while the other protein-coding genes the highest.

**Table 2:** Description statistics of *dN*, *dS*, and *dN*/*dS* in all conserved genes

Variable	Mean	Maximum	Minimum	Variance	Standard deviation
*dN*	0.7664	2.9696	0.0301	0.4097	0.6401
*dS*	4.9955	8.7323	2.1131	1.6214	1.2734
*ω*(*dN*/*dS*)	0.1445	0.5173	0.0142	0.0103	0.1013

### Characteristics of codon usage bias

**Fig. 3:**
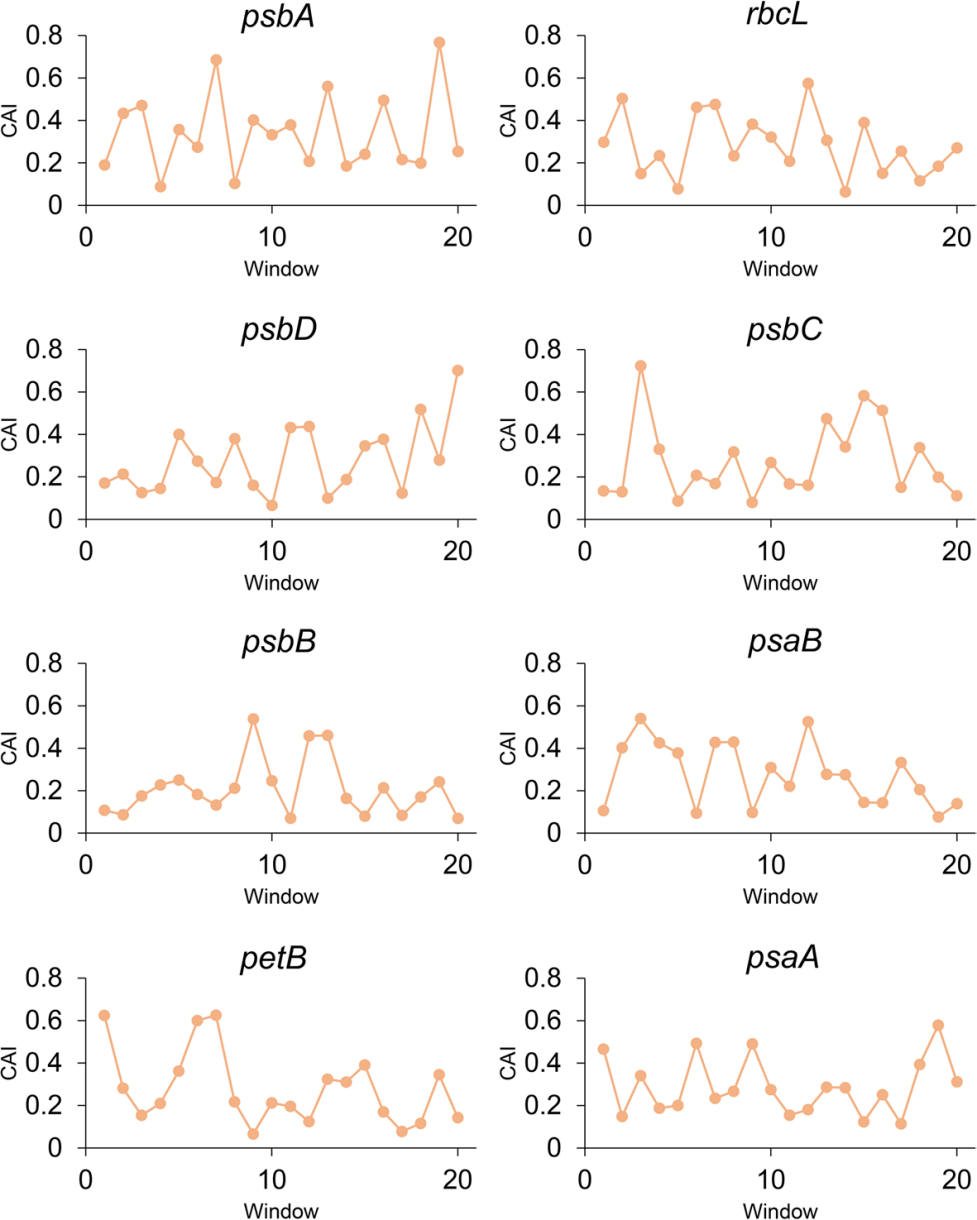
A plot of the Codon adaptation index (CAI) of the highly expressed genes (the first 20 windows). There were ten codons (in length) in every nonoverlapping windows (beginning with the 5’ end of gene sequences)

A total of 56 coding genes were obtained (Additional file 6), with sequence length ranging from 300 to 6903 bp. The average value of codon adaptation index (CAI), frequency of optimal codon usage (Fop), and tRNA adaptation index (tAI) were 0.18, 0.38, and 0.38, respectively (Table 3, Additional file 6). The Fop value ranged from 0.29 (*rpl14*) to 0.60 (*psbA*). The tAI value varied from 0.30 (*ndhD*) to 0.48 (*psbA*). Then, the *S*-value was calculated according to dos Reis et al. [39]. *O. vulgatum* was found to have a moderate *S*-value of 0.3024 [39], suggesting a moderate translational selection acting on its genome. The mean effective number of codon (ENC) value was 52.16, ranging from 41.98 (*psbA*) to 58.05 (*rpl2*). The ENC value of all genes was greater than 35, suggesting a weak codon usage bias [26]. The coefficient of variation of CAI was the largest among the indexes. Previous studies have reported a relatively low CAI value at the 5’ end of highly expressed genes [23]. Here we examined eight highly expressed genes as well (Fig. 3). The first window represents the first ten codons at 5’ end. A relatively lower CAI values was detected at the 5’ end of genes *psaB, psbA*, *psbB, psbC*, *psbD*, and *rbcL*. But no such a decrease was found at the 5’ end of *psaA* and *petB*.

**Table 3:** Descriptive statistics of CAI, Fop, ENC, and tAI

Variable	Mean	Maximum	Minimum	Variance	Standard deviation	Coefficient of Variation
CAI	0.18	0.35	0.12	0.0016	0.0395	21.9731
Fop	0.38	0.60	0.29	0.0026	0.0511	13.5103
ENC	52.16	58.05	41.98	8.3906	2.8967	5.5539
tAI	0.38	0.48	0.30	0.0015	0.0387	10.1615

The average of gene GC content was 42.35%, with the minimum 37.29% (*ycf1*) to the maximum 49.59% (*rps12*) (Additional file 6). GC content of the first, second, and third base (represented as GC_1_, GC_2_, GC_3_) was shown in Fig. 2c. And the average of GC_3_, GC_2_, and GC_1_ was 34.91%, 42.01%, and 50.13%, respectively. The GC_2_ was the closest to the total GC. The relative synonymous codon usage (RSCU) value for each codon was shown in Fig. 2d. The codon was defined as with high-frequency when RSCU > 1 [15]. We identified 29 high-frequency codons (shown in white in Fig. 2d). There were 26 codons ended in A or T, accounting for 89.66% of the total high-frequency codons.

#### Correlation between expression level and *dN*,* dS*, and *dN/dS* in chloroplast genes

**Fig. 4:**
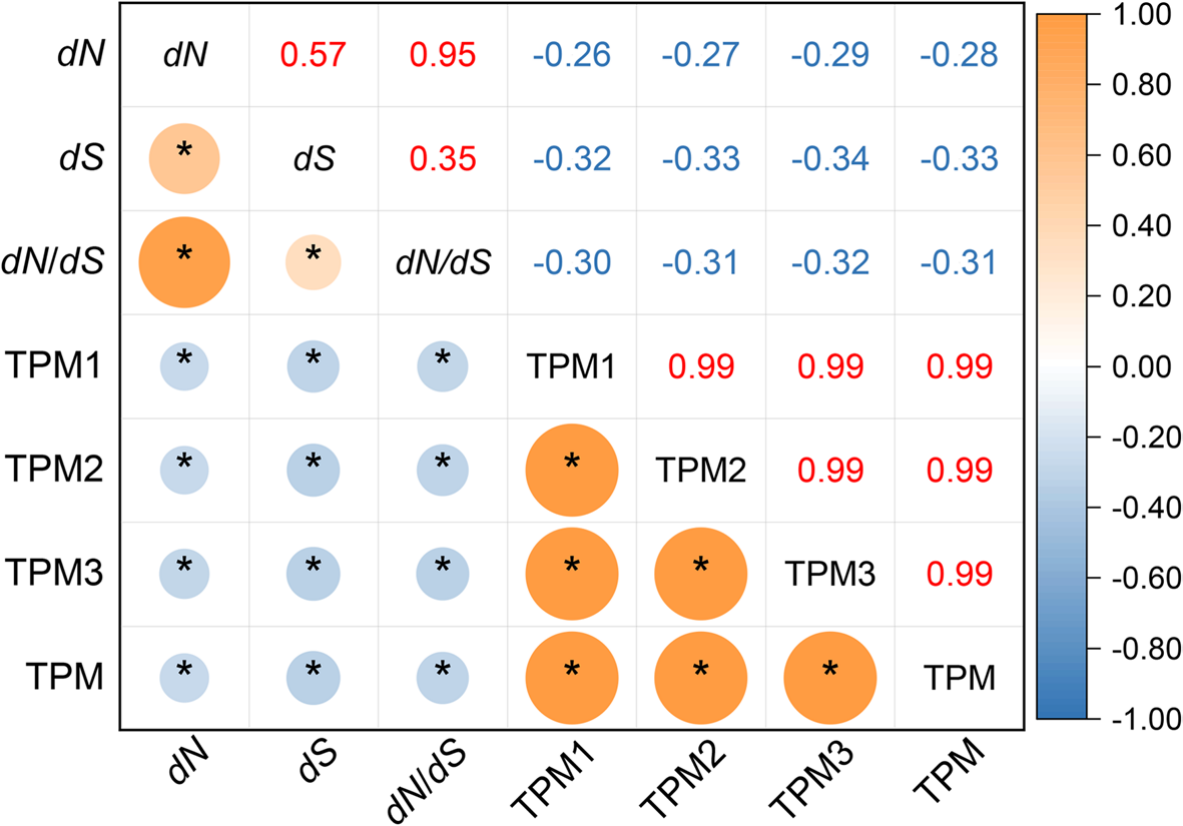
The Pearson correlation coefficient analysis results among the TPM values, *dN*, *dS* and *dN*/*dS* (64 conserved genes). The numbers on the upper triangular are correlation values. * represents significant at the 0.05 level

Correlations between TPM and *dN*, *dS*, *dN/dS* were shown in Fig. 4 (Additional file 7). The correlation coefficient of *dN* vs TPM1, *dN* vs TPM2, *dN* vs TPM3, and *dN* vs TPM was -0.264 (*P* = 0.035), -0.273 (*P* = 0.029), -0.288 (*P* = 0.021), and -0.278 (*P* = 0.027), respectively. The correlation coefficient of *dS* vs TPM1, *dS* vs TPM2, *dS* vs TPM3, and *dS* vs TPM was -0.316 (*P* = 0.011), -0.326 (*P* = 0.009), -0.336 (*P* = 0.007), and -0.331 (*P* = 0.008), respectively. Moreover, the correlation coefficient of *dN/dS* vs TPM1, *dN/dS* vs TPM2, *dN/dS* vs TPM3, and *dN/dS* vs TPM was -0.297 (*P* = 0.017), -0.306 (*P* = 0.014), -0.323 (*P* = 0.009), and -0.311 (*P* = 0.013), respectively. Of note, there was a significant negative correlation between the average value of *dN, dS,* and *dN*/*dS*, and TPM at the significance level of 0.05. Gene expression level was found to be negatively correlated with evolutionary rates and selective pressure in the cp genes of *O. vulgatum*.

#### Correlation between chloroplast gene expression level, codon usage bias and CDS length

**Fig. 5:**
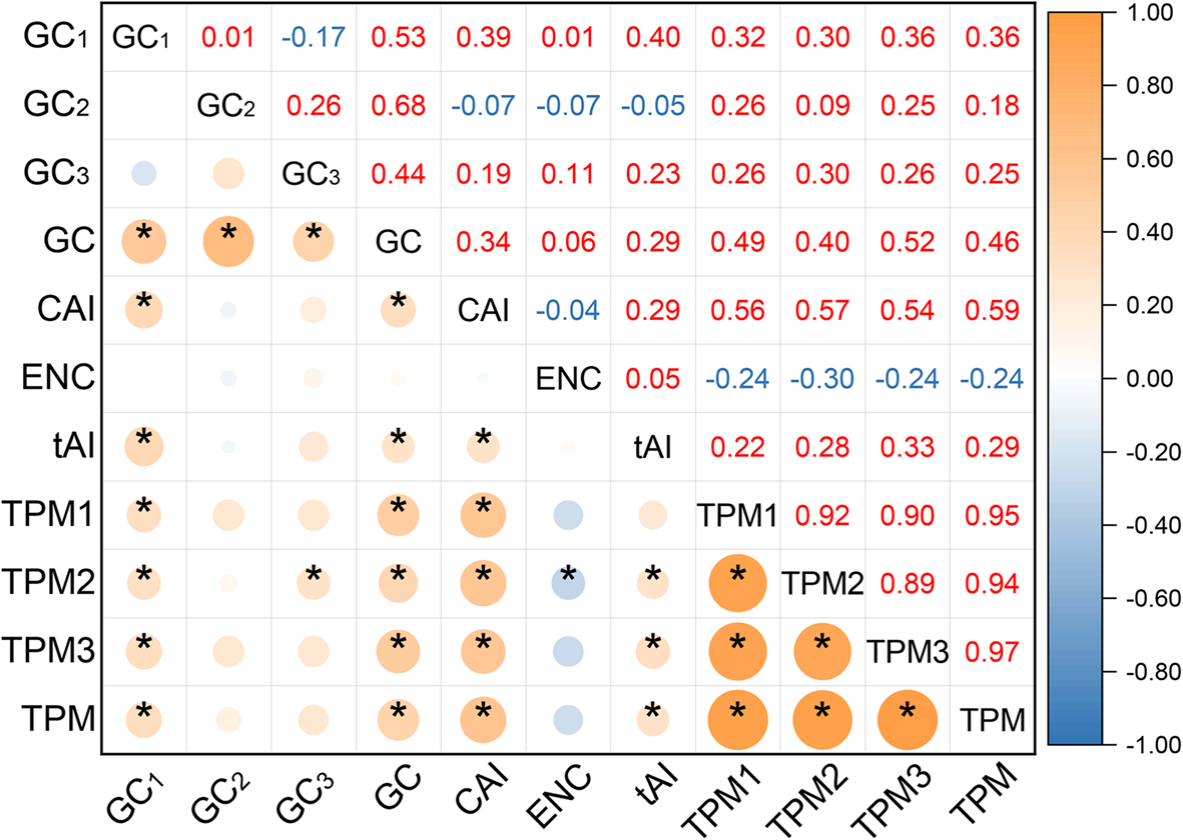
Heat map of the correlation between codon usage bias and gene expression level (56 filtered genes). Effective number of codons (ENC); the tRNA adaptation index (tAI). The numbers on the upper triangular are correlation values (Spearman’s rank correlation coefficient). * represents significant at the 0.05 level

Results of Spearman’s rank correlation coefficient indicated that the GC, CAI, and tAI exhibited a significant positive correlation with expression level (*P* < 0.05). The positive correlation coefficient (from large to small) of CAI vs TPM, GC vs TPM, and tAI vs TPM were 0.585, 0.456, and 0.287, respectively (Fig. 5). The ENC was negatively correlated with the expression level (*P* = 0.078). The correlation coefficient of ENC vs TPM was -0.237 (Fig. 5). In addition, we also calculated the Spearman’s rank correlation coefficient among gene expression level, codon usage bias and CDS length (Additional file 1, Fig. S1). The CDS-length is positive correlation with GC, CAI, Fop (frequency of optimal codons usage), TPM1, TPM2, TPM3, and TPM. Of them, only CAI value is significantly positive correlation (*P* = 0.025 < 0.05). However, the CDS-length is negatively correlated with ENC, *dN*, *dS*, and *dN/dS* but not significant. The results indicated that cp gene expression level and protein length influence the codon usage (CAI). In addition, the extent of adaptation of a gene to its genomic tRNA pool (tAI) is higher, when the expression level is higher. Gene expression level was detected positively correlated with codon usage bias in the cp genes of *O. vulgatum*.

#### Correlation of *dN*, *dS*, and *dN*/*dS* with codon usage bias

**Fig. 6:**
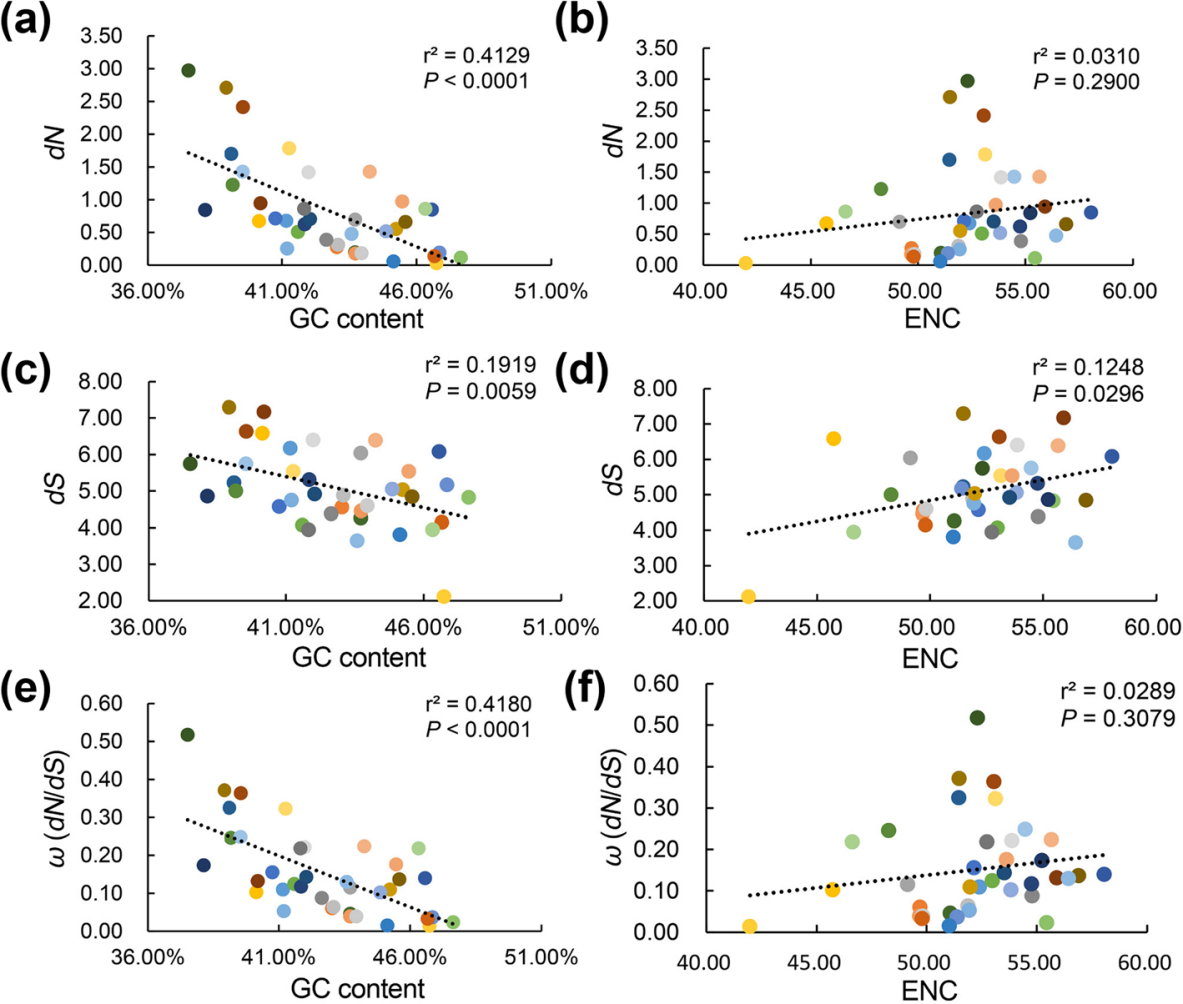
Correlation analysis of the evolutionary rates, selective pressure, GC content and ENC. **(a, c, e)** Correlation analysis between *dN*, *dS*, and *dN*/*dS*, and all GC content. **(b, d, f)** Correlation analysis between *dN*, *dS*, and *dN*/*dS*, and all ENC

We further examined whether codon usage bias is negatively correlated with the evolutionary rates. A total of 38 overlapping genes were selected from the 64 conserved genes (analyzed for evolutionary rate) and 56 genes (analyzed for codon usage bias) to test the hypothesis. Pearson correlation results indicated that the *dN*, *dS*, and *dN*/*dS* had a significant negative correlation with GC content (*P* < 0.001) (Fig. 6a, c, e). The *dN*, *dS*, and *dN*/*dS* exhibited a positive correlation with ENC but not significant except for *dS* (Fig. 6b, d, f). Briefly, when the ENC is lower, the codon usage bias tends to be stronger, the nonsynonymous substitution rate and selective pressure be lower, but the correlation is not significant.

## Discussion

### Expression level and *dN, dS,* and *dN*/*dS* are negatively correlated in chloroplast protein-coding genes

We detected a negative correlation between the average TPM values and *dN*, *dS*, and *dN/dS*, of the cp genes in *O. vulgatum*. This is consistent with the findings in *Brassica* [6], *Arachis* [7], and *Pyrus* orthologous genes [8] as well as *Brassica napus* vernalization-pathway genes [3]. These results further consolidate the E-R anticorrelation relationship between gene expression level and evolutionary rates [4, 5]. To explain the anticorrelation, Drummond and Wilke [40] proposed that protein misfolding induced by mistranslation represents the main constraint on the evolution of protein-coding sequences. Gout et al. [41] constructed a COSTEX model based on the trade-off between benefit and cost of the expression level. The model predicts that selective pressures against mutations leading to changes of gene expression level or protein encoding on average are stronger in highly expressed genes; and, as a result, the protein evolutionary rate appears negatively correlated with its gene expression level. Similarly, Cherry [2] postulated that more highly expressed proteins tend to be under stronger selection; namely, selection imposes a greater restriction on their sequence changes and lower the evolutionary rate. Moreover, other hypotheses have been proposed as well: (1) the avoidance of false protein interactions leads to a slow evolution of highly expressed proteins [42], i.e., the protein misinteraction avoidance hypothesis; (2) the need for stronger mRNA folding leads to a slow evolution of higher expressed genes and proteins [43], i.e., the mRNA folding requirement hypothesis. However, Feyertag et al. noticed that the E-R anticorrelation is not always effective for secreted proteins [44]. As there exist one inner membrane protein and five transmembrane proteins encoded by cp genes in *O. vulgatum* [38], we also examined their expression level—evolutionary rate correlation. Our results are consistent with the E-R anticorrelation prediction but not statistically significant. Taken together, the present study indicates that the E-R anticorrelation is effective for cp protein-coding genes, but more mechanistic details remain to be characterized.

### Expression level and codon usage bias are positively correlated in chloroplast protein-coding genes

A positive correlation between gene expression level and codon usage bias had been noted in *Escherichia coli* and *Saccharomyces cerevisiae* in previous studies [45]. Higher cp gene expression with stronger codon usage bias has been detected in *Hordeum vulgare*, *Triticum aestivum*, *Oryza nivara* and other five species in Arundinarieae [46]. In this context, it is expected that gene expression levels may affect cp genome codon usage as well [46]. Here we have observed a significant positive correlation between the cp gene expression level and CAI and tAI in *O. vulgatum*. Namely, the higher the gene expression, the larger the adaptation extent of a gene to its tRNA pool, and the stronger preference for codon usage bias. This may be caused by the optimization of codon usage driven by translation, because the codons of highly expressed genes impose greater impact on translation efficiency [47]. How does codon usage bias affect translation efficiency? There have been two hypotheses suggesting that: (1) high-frequency codons are selected because this may reduce the probability of being recognized by non-matched tRNAs during translation (tRNAs encoding preferred anticodons lead to more precise amino acid acylation). As a result, this may decrease the production of error proteins and enhance the functional protein synthesis rate [20, 40, 48]. And (2) high-frequency codons are chosen because they decode rapidly, increasing the translation efficiency [48, 49]. Moreover, codon usage bias may change mRNA levels by affecting mRNA splicing [50] and mRNA stability [51]. Presnyak et al. [51] have evaluated the contribution of each codon to RNA stability independently. They found that optimal codons are enriched in mRNA with a long half-life. This study shows that the frequency of high-frequency codons is positively proportional to gene expression level. The patterns of codon usage bias implies that synonymous mutations are not always truly silent, and they may have a function in finely regulating gene expression [48]. It has been noted that making the rare codon usage in target gene similar to the host codons enable to increase expression without modifying amino acid sequence [52]. Additionally, our results highlight that protein length has a positive correlation with codon usage (CAI), which is in line with the findings of *Escherichia coli* nuclear genes [24]. To explain this correlation, it has been suggested that the longer length may facilitate to improve the accuracy of selecting optimal codons [24]. Nevertheless, conversely, Ingvarsson reports a negative correlation between protein length and codon usage bias in *Populus tremula* nuclear genes [16]. We speculated that the concordance of cp genes and *E. coli* may be related to the prokaryotic origin of chloroplast [53].

### Codon usage bias in *O. vulgatum* cp genes

Our results showed that the A/T content at the third position of *O. vulgatum* is greater than G/C content. Of the 29 high-frequency codons, 26 are identified ended in A/T. Generally, the third position tends to bias towards ending with A/T for cp codons. For instance, Zhou et al. [54] have observed the tendence in *Arabidopsis thaliana*, *Populus alba*, *Zea mays*, *Triticum aestivum*, *Pinus koraiensis*, and *Cycas taitungensis*; Wang et al. [55] in six Euphorbiaceae species, and Duan et al. [26] in *Delphinium grandiflorum.* But for nuclear genes, Kawabe and Miyashita [56] find that G/C is preferred at the third position in four monocot species. This suggests that codon usage bias could be different in different genomes and also reflects the independent evolution of cp genome from nuclear genome. The average GC content of *O. vulgatum* is GC_1_ (50.13%) > GC_2_ (42.01%) > GC_3_ (34.91%); GC_3_ content is the smallest, and the composition of the second codon position shows similarity to the overall composition. The average GC content of the first codon is within the upper and lower quartiles, while that of the third codon being the lowest. Similar results have been reported in cp genes of *Triticum aestivum* [14], *Hemiptelea davidii* [15], and *Delphinium grandiflorum* [26]. Noteworthily, the cp gene expression level of *O. vulgatum* shows a significant positive correlation with GC_3_ and GC. As G/C forms three hydrogen bond pairs, higher GC content may increase the binding strength and improve translation efficiency [15].

Previous studies have shown a relatively low CAI value at the 5’ end of highly expressed genes [23]. This is also the case in *O. vulgatum* highly expressed genes (Fig. 3). The first 10 codons at the 5’ end of *psaB, psbA*, *psbB, psbC*, *psbD*, and *rbcL* have a relatively lower CAI values. The mRNA secondary structure corresponding to the 5’ end of a gene can indirectly influence the codon usage frequency [22]. It has been suggested that selection of 5’ end codon usage may lead to increase initiation rate or affect expression [23, 57]. But no such a decrease of CAI values occured at the 5’ end of *psaA* and *petB*. We notice that the *dN*/*dS* value of *psaA* and *petB* is higher than that of other 6 highly expressed genes. However, the association between CAI and evolutionary rate remain to be determined.

## Conclusions

In conclusion, we have used cp genome and transcriptome data in *O. vulgatum* to reveal the correlations between cp gene expression level and *dN, dS* and *dN*/*dS*, and codon usage bias. Our results demonstrate that cp gene expression level is negatively correlated with evolutionary rates and selective pressure, but positively correlated with codon usage bias and tRNA adaptation index. This provides novel evidence consolidating the E-R anticorrelation relationship between expression level and evolutionary rate.

## Materials and methods

### Plant materials

All plant materials were collected from South China Agricultural University (E113°20', N23°9'). Voucher specimens were stored in the Herbarium of South China Agricultural University (SCAUB, the voucher IDs were shown in Additional file 8). The unique morphology of the sporophyte of *O. vulgatum* is composed of a single vegetative leaf and a sporophyll with sporangia spikes (Fig. 1a). Leaves of a total of 60 individuals were collected under the same growing conditions at the same age. The collected leaves were immediately immersed in liquid nitrogen and stored at -80 degrees until RNA extraction. They were divided into three groups, each of which was a biological replicate.

### RNA extraction, cDNA library construction and sequencing

RNA extraction was performed using Trizol Reagent (MagZol™ Reagent, R4801-03, China). After extraction, the RNA samples are stored at -80 ℃ for library construction. RNA was quantified and quality evaluated using the Nanodrop (Thermo Fisher Scientific, USA) and an Agilent 2100 Bioanalyzer (Agilent Technologies, USA). The mRNA was enriched by Oligo (dT) magnetic beads and broke into fragment under the action of high temperature and metal ions. Random hexamers were used to synthesize the first cDNA chain and followed by adding enzymes, buffer, dNTP mixture (dATP, dTTP, dGTP, and dCTP) to synthesize the second cDNA chain. Finally, the synthesized double-stranded cDNA was purified by magnetic beads. The end was repaired and A was added to connect the sequencing connector, and the fragment size was sorted by using magnetic beads. The sorted fragments were enriched by PCR, and the PCR products were purified to construct the final library. Illumina Novaseq6000 (Illumina, USA) high-throughput sequencing platform (Science Corporation of Gene, Guangzhou, China) was used to sequence libraries. The sequencing strategy was PE150 (Pair-End 150), and the amount of sequencing data of each sample was not less than 6 Gb clean reads.

### TPM calculation of chloroplast genes

Our sequenced cp genome has submitted in NCBI (GenBank number MZ066610). The gene reads were extracted with the PhyloSuite Version 1.2.1 [58] software. We have used a custom Perl script to run the RSEM software [59] for quantifying read counts from RNA-seq (the Perl Script was deposited in the GitHub at https://github.com/yy-liang/TPM_Calculator). Finally, the TPM value of all genes was calculated. The Correlation Plot APP of OrginPro 2021b was used for plot the correlation heat map (the same as below).

### Analysis of *dN*,* dS*, and *dN*/*dS* of chloroplast genes

We downloaded 12 published ferns cp genome sequences from NCBI (Table 4) to analyze the evolutionary rates and selective pressure. The CDS sequences of common cp genes of *O. vulgatum* and the 12 ferns were extracted by PhyloSuite Version 1.2.1. Duplicated genes and non-conserved gene sequences were deleted. Muscle (Codons) in MEGA7.0 [60] was used for sequence alignment and with manual correction. The codeml program in PAML4.9 [61, 62] was used to calculate *dN*, *dS*, and *dN*/*dS* using ML method (the trees file was generated by using PhyloSuite to construct the Bayesian phylogenetic tree [58, 63], Additional file 3). Descriptive statistics were calculated by using SAS (SAS Institute Inc., Cary, NC, USA) software. The R-plugin in TBtools [64] was used for plot the correlation map (the same as below). OrginPro 2021b was used to draw the boxplot (the same as below).

**Table 4:** Ferns name and their GenBank accession numbers

Fern name	Genbank accession number
*Lepisorus clathratus*	KY419704
*Leptochilus hemionitideus*	MH319943
*Athyrium anisopterum*	KY419703
*Diplazium bellum*	KY427343
*Alsophila spinulosa*	FJ556581
*Cyathea lepifera*	MN623357
*Actinostachys pennula*	KU764518
*Schizaea elegans*	KX258660
*Ophioglossum californicum*	KC117178
*Botrychium lunaria*	MN966674
*Mankyua chejuensis*	KP205433
*Equisetum arvense*	JN968380

### Analysis of codon usage bias

The cp protein-coding gene sequences of *O. vulgatum* was extracted. In order to eliminate sample bias, the coding sequences with a length less than 300 bp were filtered out [65]. Due to the large amount of RNA editing in the protein-coding genes of ferns, we also performed manual correction. Finally, the complete protein-coding sequences were used for subsequent analysis (all analysis excluding stop codons). CodonW 1.4.2 [66] was used to calculate CAI, Fop, ENC, and RSCU automatically. Then codon was defined as with high-frequency when RSCU > 1 [15]. In order to understand whether a relatively low codon adaptation occurs at the 5’ end of highly expressed genes. We divided each highly expressed genes into nonoverlapping windows (ten codons in length, beginning with the start codon, 5’ end) according to the method of Morton et al. [23]. Then CAI value was calculated for each window separately. R 3.6.3 software and Perl script were used according to the method of dos Reis et al. [39] to calculate the tAI and *S*-value. The tAI estimates the extent of adaptation of genes to their tRNA pool [67]. *S*-value explains selection on codon usage in genomes, higher *S*-value indicates stronger action of translational selection due to tRNA adaptation (between -1 and 1). Descriptive statistics of each value were calculated by using SAS. The CUSP program of EMBOSS software [68] was ran to calculate the GC content of codons, GC_1_, GC_2_, and GC_3_.

### Correlation analysis

The value range of correlation coefficient r is -1 ≤ r ≤ 1. When the significance level is greater than the probability *P* value, the two variables are considered to be correlated. IBM SPSS—version 19.0 was used to calculate Spearman’s rank correlation coefficient and the CORR program in SAS software to calculate the Pearson correlation coefficient (among the molecular evolutionary rates, gene expression level, codon usage bias, and CDS length).

## Supplementary Information

**Additional file 1. Figure S1. **Heat map of the correlation among the CDS-length, evolutionary rates, selective pressure, and gene expression level. The numbers on the upper triangular are correlation values (Spearman’s rank correlation coefficient). * represents significant at the 0.05 level.

**Additional file 2. Table S1. **TPM values of all genes.

**Additional file 3. Trees file: **Phylogenetic tree file.

**Additional file 4. Table S2. ***dN*, *dS*, and *dN*/*dS* values of all conserved genes.

**Additional file 5. Table S3. **The type of 64 conserved genes of ferns.

**Additional file 6. Table S4. ** Codon usage bias of all genes.

**Additional file 7. Table S5. **Pearson correlation analysis between *dN*, *dS*, and *dN*/*dS*, and expression level.

**Additional file 8. Table S6. **Voucher information of three biological replicates in *Ophioglossum vulgatum*.

## Data Availability

The transcriptome data were deposited in NCBI Sequence Read Archive (SRA) at (https://www.ncbi.nlm.nih.gov/bioproject), the accession number is PRJNA789441.

## Abbreviations

cp: Chloroplast
*dN*: Nonsynonymous substitution rate
*dS*: Synonymous substitution rate
*dN*/*dS*: Selective pressure (omega, *ω*)
TPM: Transcripts Per Million
CAI: Codon adaptation index
tAI: tRNA adaptation index
E-R anticorrelation: An anticorrelation relationship between gene expression level and evolutionary rate
tRNA: Transport RNA
mRNA: Messenger RNA
NCBI: National Center for Biotechnology Information
SRA: Sequence Read Archive
Q20: The ratio of bases with quality values greater than 20 (less than 1% error rate) to total bases
Q30: The ratio of bases with quality values greater than 30 (less than 0.1% error rate) to total bases
CDS: Protein coding sequence
Fop: Frequency of optimal codon usage
ENC: Effective number of codon
RSCU: Relative synonymous codon usage
dNTP: Deoxy-ribonucleoside triphosphate
cDNA: Complementary DNA
PCR: Polymerase chain reaction
PE: Pair-End
