# Supplementary figures and images for: Chloroplast gene expression level is negatively correlated with evolutionary rates and selective pressure while positively with codon usage bias in Ophioglossum vulgatum L
Source: BMC Plant Biol. 2022 Dec 13;22:580. doi: 10.1186/s12870-022-03960-8 (PMC9746204; doi:10.1186/s12870-022-03960-8)

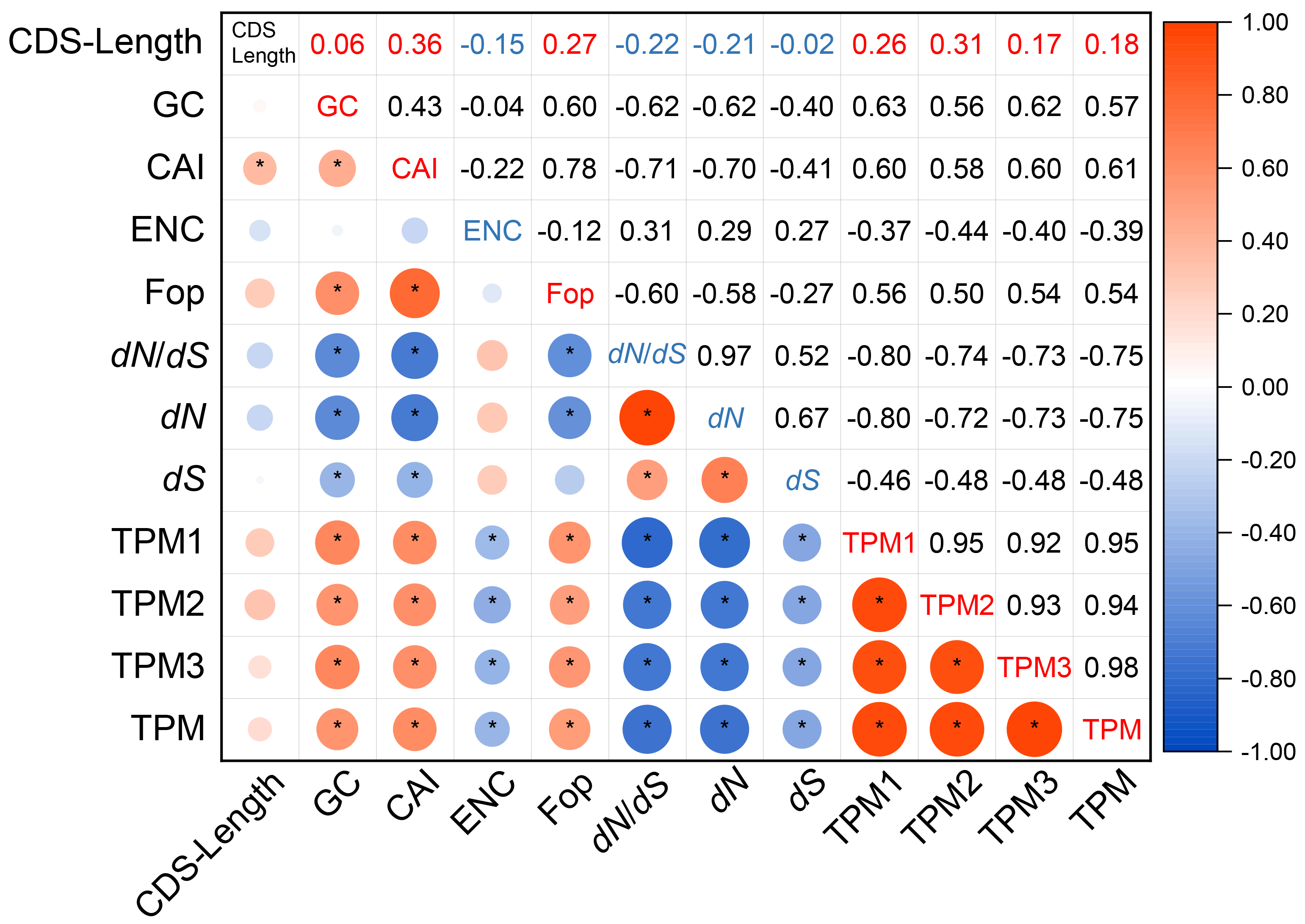

Supplement: Supplementary file 1 — Additional file 1. Figure S1. Heat map of the correlation among the CDS-length, evolutionary rates, selective pressure, and gene expression level. The numbers on the upper triangular are correlation values (Spearman’s rank correlation coefficient). * represents significant at the 0.05 level. [file 12870_2022_3960_MOESM1_ESM.jpg]
